# The role of heparan sulfate deficiency in autistic phenotype: potential involvement of Slit/Robo/srGAPs-mediated dendritic spine formation

**DOI:** 10.1186/s13064-016-0066-x

**Published:** 2016-04-18

**Authors:** Christine Pérez, Darrell Sawmiller, Jun Tan

**Affiliations:** Rashid Laboratory for Developmental Neurobiology, Silver Child Development Center, Department of Psychiatry and Behavioral Neurosciences, Morsani College of Medicine, University of South Florida, 3515 E Fletcher Ave., Tampa, FL 33613 USA

**Keywords:** Autism spectrum disorders, Heparan sulfate, Robo, Slit, srGAPs, Glycosoaminoglycans, Dendritic spines, Axonal guidance, Neurogenesis, Sulfotranferases

## Abstract

Autism Spectrum Disorders (ASD) are the second most common developmental cause of disability in the United States. ASDs are accompanied with substantial economic and emotional cost. The brains of ASD patients have marked structural abnormalities, in the form of increased dendritic spines and decreased long distance connections. These structural differences may be due to deficiencies in Heparin Sulfate (HS), a proteoglycan involved in a variety of neurodevelopmental processes. Of particular interest is its role in the Slit/Robo pathway. The Slit/Robo pathway is known to be involved in the regulation of axonal guidance and dendritic spine formation. HS mediates the Slit/Robo interaction; without its presence Slit’s repulsive activity is abrogated. Slit/Robo regulates dendritic spine formation through its interaction with srGAPs (slit-robo GTPase Activating Proteins), which leads to downstream signaling, actin cytoskeleton depolymerization and dendritic spine collapse. Through interference with this pathway, HS deficiency can lead to excess spine formation.

## Background

Autism Spectrum Disorders (ASD) are a set of neurocognitive developmental disorders that are associated with substantial deficits in social interaction. It is the second most common developmental disability in the United States, with approximately 16 out of 10000 people amongst the general population and 14.7 out of 1000 amongst children under eight having an autism spectrum disorder [1–3]. Amongst children under eight have autism there is a higher prevalence of autism amongst boys than girls (one in 42 boys, one in 189 girls) [3]. The associated cost of autism in the United States is approximately $2.4 million [4]. With the stark increase in the prevalence of ASD (269 % from 1996 to 2010) and the associated cost of treatment, there has been an increased interest in the study of ASD pathophysiology [5]. Amongst the many potential causes of ASD, deficiencies in Heparan Sulfate (HS) have garnered interest.

## Autism

Along with the social deficits displayed in ASD, substantial structural changes are observed in the autistic brain. Autistic infants have normal brain size at birth, but following birth their brains grow faster than normal [6]. After the first critical period in postnatal brain development, the brains of autistic patients were 10 % larger [6]. Conversely, during the second and third period of growth, at ages 2–5 and 5–11, respectively, the brains of autistic patients experienced delayed growth. Along with these macrostructural differences, there are also microstructural differences in ASD brains. These differences include disturbances in axons and dendrites as well as in the number of neurons present. The cerebrum of ASD patients had excess cortical neurons when compared to the non-ASD brain. In contrast, the cerebellum displayed a lower number of neurons, in particular Purkinje cells. The white matter of the cerebrum also exhibited abnormalities, such as thin axons and less myelinated axons than controls [7]. Both of these morphological changes are associated with increased short distance connections, accompanied by decreased long distance connections. The white matter of ASD patients displayed high levels of GAP-43 (growth associated protein-43), a substance associated with cell proliferation, suggesting excess neurogenesis. This supports past findings of excess neurons in the cerebrum of patients with ASD.

Various abnormalities in dendrite morphology are observed in the autistic brain. For a normal child the frontal cortex does not reach its full level of dendritic arborization until the end of childhood, with 3 % of its size present at birth and only 48 % present at 2 years [6]. The cerebral cortex develops more slowly than other brain areas [6]. The extended time it takes for the frontal cortex to develop makes it especially vulnerable to disruption. One study on postmortem brain tissue showed significant differences between the dendritic spines of ASD brains and controls [8]. ASD brains had a higher average density with the difference most apparent in the second layer. Layer V also had a significant relationship with diagnosis, with ASD brains having higher densities in the temporal lobe. In addition to the increase in spine density, shorter spines were also observed in ASD brains. There was also notable differences in the type of dendritic spines present. There were more oblique spines in ASD patients than in controls. The oblique spines also had a greater spine density than either apical or basilar spines did within ASD brains, in controls the spine densities were more similar across type. The density of apical spines within Layer II was found to be higher in ASD subjects than in controls [8].

Exposure to valproic acid, an anticonvulsant in utero has been linked to autism and other cognitive deficits [9, 10]. A study on rat embryos exposed to valproic acid has led to insight into the structure of ASD brains. Pregnant rats were exposed to valproic acid at gestational day 11.5 [9]. The rats were sacrificed 2 weeks after birth and the microcircuitry of the rats was examined. Synapses showed reduced maximum activity and a weaker excitatory synapse response. The experimental group had an average maximum synaptic output of 3.3 ± 0.3 mV compared with an average of 4.2 ± 0.4 mV for control synapses. Greater current was needed to produce an action potential for Layer V neurons 400 ± 16 pA, *n* = 51 were needed for the control group, and 444 ± 16 pA, *n* = 31 for the experimental group. There was also a possible increase in local wiring. The number of direct connections increased 50 % at short distances (~50 μm), while there was no difference for long distance connections (100–200 μm) [9]. There was also an increased probability of interneuron connections. Accompanying this increase there was hyperactivity at the local level with an increase in the synaptic release of excitatory neurotransmitters. In all there appeared to be more connections and neurotransmitter release, but weaker signals and communication.

Together, these findings suggest that the ASD brain never progresses past the early part of brain development, which is characterized by excess neurogenesis and immature synaptic connections. While the structural changes in the ASD brain have been characterized, it is unclear what causes these changes. There is some suggestion that Heparan Sulfate deficiencies may play a role.

## Heparan sulfate

Heparin Sulfate (HS) is a proteoglycan that has a variety of biological roles. Proteoglycans are made of chains of highly charged glycosaminoglycans (GAGs). There are five classes of HS which include: membrane syndecans, glycosyl-phosphatidylinositol-anchored (GPI-anchored) glypicans, perlecan, agrin, and hybrid HSPG/collagen type XVIII [11]. Most of the cell-surface HS is in the form of syndecans, which are integral membrane proteins [12]. There are six glypicans in vertebrates, and glypicans may play a role in growth factor signaling pathways [11, 12]. Perlecan and agrin are two of the three proteoglycans of the basement membrane [13]. Perlecan is important in cell differentiation and embryogenesis [13]. Agrin is the main proteoglycan at the neuromuscular junction and causes the aggregation of acetylcholine receptors [13].

HS is formed in a multiple step process which includes: the formation of a membrane bound protein core (in the case of membrane bound HS), initiation/elongation of the GAG, and various sulfation or de-acetylation steps. HS synthesis is a highly evolutionarily conserved synthesis pathway consisting of 14 steps [11, 12]. The process requires several enzymes making it vulnerable to mutations, being that a mutation of one enzyme could prevent subsequent steps. The first step, mediated by peptide-O-xylosyltransferase, initiates the GAG chain on the protein core [14]. The GAG chain constituents themselves are formed in the Golgi apparatus by various enzymes [15]. HS GAG chains consist of repeats of *2* N-Acetylglucosamine (GlcNAc) units and 1 Glucuronic acid (GlcA) unit [15]. The 2 GlcNAc units are added to the chain by galactosyltransferases-I and –II and the GlcA is added by Glucuronyltransferase I [15]. GlcA is donated by UDP-glucuronic acid which is produced by UDP glucose dehydrogenase (UDPGDH) [12]. Continued elongation of the GAG chain is accomplished by exostosin (EXT)-family co-polymerases (EXT1, EXT2, EXTL1, EXTL2 and EXTL3), a family of proteins with highly conversed C-terminals, which are thought to be involved in HS synthesis [14–16]. A heteroligomeric complex of EXT1 and EXT2 polymerizes the GAG chain in HS, the roles of EXTL1, EXTL2, EXTL3 are still unknown, although sequence homology suggest that there function is similar to that of EXT1 and EXT2 [14–16]. Once elongation is completed HS chains are typically ∼ 5 to ∼ 70 kDa in GAGs attached to the protein core [11]. Following the formation of the GAG chain, the proteoglycan undergoes several modifications including: C5 epimerization of GlcA, de-acetylation and multiple sulfation [14, 15]. The C5 epimerization of GlcA is mediated by heparan sulfate C5 epimerase (HS C5-EP) [15]. The multiple sulfation steps lead to many different patterns, which are regulated in a cell specific way [15]. The sulfation patterns of HS modify cellular activity and signaling in different ways, such as providing different axonal guidance cues [14]. The multiple sulfation steps are mediated by several different enzymes: HS 2-O-sulfotransferases (H2ST) which transfers sulfate to GlcA/IdoA residues, 6-O-sulfotransferases (H6ST-1, −2, −2S and −3) which catalyzes GlcN 6-O-sulfation, and 3-O-sulfotransferases (H3ST-1, −2, −3A, −3B, −4, −5 and −6) which catalyzes GlcN 3-O-sulfation [12, 15].

The biological roles of HS have been known to include the mass migration of cells, the protection of FGF from proteolysis, and the formation of the extracellular matrix (ECM) [12, 17]. It is well known that HS has a role in neural development, through the modulation of neurogenesis, axonal guidance, and synaptogenesis [18]. HS has regulated neural connectivity since cnidarians and it is thought to have appeared around the time that the first nervous systems did [14, 19]. This suggests that HS is necessary for proper neural development. Its role in the protection of FGF-2 is critical to neurogenesis, since FGF-2 is a critical growth factor in neural stem cell (NSCs) differentiation and proliferation [18]. Also glypican-4 (K-glypican) is highly expressed in the developing brain of embryos [20]. Furthermore, glypican-4 is expressed primarily in the ventricular zone of the telencephalon, suggesting that it plays a role in cerebral neurogenesis [21]. Glypican-4 was found to be expressed with nestin, a marker for neural progenitor cells, further suggesting its role in neurogenesis [21]. Perlecan also interacts with FGF-2 by acting as its co-receptor [18]. Syndecan-3 has been found on growth cone surfaces and implicated in axonal guidance [18]. Syndecan-3 also interacts with the post synaptic protein CASK, possibly contributing to synapse formation [15, 18]. HS also plays a role in the activity of neurotropic factors, ECM, cell adhesion molecules (CAMs), morphogens, and chemotropic factors [14]. HS has also been shown to facilitate axonal growth, but the associated increase of HS at the sight of injury has been shown to inhibit axonal regrowth [14]. HS is necessary for Slit, Ephrin-A3, and Semaphorin 5A axonal guidance signal pathways [14, 15]. It is thought that the sulfation of HS creates a code that modifies the activity of these signaling pathways [14]. Furthermore, Syndecan-2, another type of HS, has been linked to dendritic spine maturation [22]. HS’s role in neurodevelopmental processes has made it of interest in the study of neurodevelopmental disorders, in particular autism.

HS deficiencies have been linked to autism [23]. In one study, conditional EXT knockout (KO) mice were shown to have many of the stereotyped behaviors associated with autism [23]. The conditional KO did not have EXT in neurons, an enzyme vital in the polymerization of HS, making the conditional KO mice HS deficient [23, 24]. The HS deficiency was at its greatest in the amygdala and hippocampus, regions important to memory and leaning [23]. The brains of the mice showed no structural, tract abnormalities or myelination abnormalities observed in ASD brains [7, 23]. There was also no difference in dendritic spines as is typical with ASD [7, 23]. This can be explained by the late onset nature of the conditional knockout [23].

There were no observed motor function deficits, but there were significant social deficits observed [23]. EXT KO mice displayed reduced nest building, a stereotyped behavior associated with autism. The mice also showed behavior associated with autism during the Separation-reunion test, Resident-intruder test, Social dominance tube test, and Holed-board test. The KO mice had ultrasonic vocalizations (USV) that were reduced in number, amplitude, and duration. The mice also had hypersensitivity to a hot plate. Mapping of neuronal activity was conducted with Neuronal c-Fos induction as an activity marker. Activity was found to be lower in the basolateral and medial amygdala as well as the ventral orbitofrontal cortex of KO mice when compared to controls. Similar to other autism studies, the KO mice brains showed inhibited synaptic response to excitatory stimuli [9, 23]. This decrease in response could be due to the associated decreased level of AMPA [23]. Furthermore, two brothers with mental retardation associated autism, were shown to have EXT mutations [25]. Suggesting disruptions in EXT mediated GAG chain elongation can play a role in ASD pathogenesis. In addition, these studies suggest the social behavioral abnormalities associated with HS deficiency can begin long before morphological changes are noticeable.

The BTBR mouse model of autism has shown a reduction in HS associated in fractones in the subventricular zone (SVZ) [26]. The number of fractones in the control brain was an average of 1080 for two brains, while the BTBR mice had an average 345 for two brains. Along with the reduction in HS fractones there were noticeable structural differences. Agenesis of corpus callosum was observed as well as reduced size of latent ventricular cavities. There was a 40 % decrease in the length of the SVZ measured in the midline of the HS-ir, suggestive of a decrease in neurogenesis.

In the brains of autistic individuals HS was deficient in the Hypocellular Layer (II) region of the SVZ of the lateral ventricle (LV-SVZ) [27]. It is worth noting that this deficiency disappeared with age. This lack of HS deficiency in older ASD brains may be explained by the natural decrease in HS in the aging brain. The brains of 5–6 years old ASD patients showed an increase in cell proliferation. This seems to contradict research on BRBR mice suggesting decreased neurogenesis, but is consistent with other postmortem studies on human brain tissue which suggest that early in development the ASD brain undergoes excess neurogenesis [6, 26, 27]. The increased neurogenesis was colocalized with the HS deficiency, suggesting HS can decrease neurogenesis [27]. The discrepancy between studies could have to do with the differences in HS levels. The colocalization of GFAP with neurogenesis is consistent with the presence of Type I progenitors. The cell proliferation was localized with the HS deficiency, implying that HS inhibits neurogenesis. The increased neurogenesis dissipated with age, which is curious being that HS deficiency also dissipated with age.

It has also been shown that ASD patients have a sulfate metabolism impairment as evident by reduced levels of sulfates in the plasma of ASD [19]. One study on 38 ASD participants and greater than 25 age matched (ages 2–16) controls, showed that along with reduced levels of plasma sulfate ASD patients also showed reduced transulfation metabolites such as: taurine, glutathione and cysteine [28]. The average total plasma sulfate of ASD patients is 934 ± 252 μmol/l versus an average of 1,930 ± 184 μmol/l for controls [28]. ASD patients were also shown to have increased plasma oxidative glutathione, which is indicative of cell damage [28]. In addition to having low plasma sulfate levels, sulfate was excreted at a higher level in ASD individuals: 6819.0 nmol/ml and 3030.8 nmol/ml in controls [29]. This is of particular interest because peptidoglycan sulfation patterns affect HS activity and function [19, 30]. One type of HS sulfotranferase, Phenol Sulfotransferase, has been linked to autism [31]. An autism genetic study found an abnormality on chromosome 16p12.1-11.2, the location of the Phenol Sulfotransferase gene [31]. Fluorescence in situ hybridization (FISH) detected a microdeletion of 16p11.2 that was not present in any of the controls [32]. A second study found that deletion or duplication of 16p11.2 is found in 1 % of those with autism and 1.5 % of those with developmental delays [33]. Along with excess sulfate excretion in patients with ASD there is also evidence of a threefold or greater increase of GAG excretion in ASD patients [34]. This points to HS defiency as a potential cause of autism.

It is unclear how a deficiency in HS can lead to autism, but it is known that HS has a role in the early development of the nervous system. HS is involved in synaptogenesis, axonal guidance, neural migration, and dendritic spine formation in the developing brain [14, 18, 35]. It appears that axonal guidance disruptions and dendritic spine deformation are the most likely candidates, because both have been shown in autism cases [36, 37]. This along with the HS deficiencies in rat models of autism makes the role of HS in the regulation of these processes a good target for autism research.

## Slit/Robo

One way in which HS regulates these processes is through its interaction with the Slit/Robo signaling pathway. Slit/Robo regulates thalamocortical projections, forebrain commissure formation, axonal guidance, and dendritic spine formation [38–40]. Robo is a cell-surface receptor and Slit is one of its ligands. Robo is a CAM in the immunoglobulin super family [41]. There are four members of the Robo family present in mammals, all of which have five immunoglobulin-like (IG) domains and three fibronectin-like repeats [42–44]. IG1 and IG2 of the five IG domains have been shown to be necessary for Slit/Robo interaction [42]. Slit is known for its four leucine-rich repeat (LRR) domains located in the N-terminus, which are labeled D1-D4 [42]. Studies on Slit have shown that Slit can function without the C-terminus in vitro; hence, the LRR domain is necessary for Slit/Robo signaling while the C-terminus is not, but this has yet to be tested in vivo [42]. The Slit/Robo interaction is a high affinity interaction [42]. Much work has gone into understanding this interaction. Through work with xenopus it was found that the highly conserved D2 domain is the binding site for Robo [42]. It appears that the D2 domain of Slit binds with the IG1 domain of Robo [42]. The residues that mediate this interaction are Thr-74, Phe-114, and Arg-117 of IG1 [45]. While the IG2 domain of Robo is not the binding site of Slit, it appears to be necessary for the proper folding of the IG1 domain [42].

HS has been shown to affect Slit’s affinity to Robo [41, 46]. In one study, HS was removed from the cell surface of Robo1 expressing cells and Slit2 binding was reduced by threefold [46]. It was also found that the repulsive effects of Slit2 on the cellular projections was abolished in vitro when cell surface HS was removed by heparinase III [46]. Slit’s interaction with HS is mediated by the D4 domain of Slit [47]. The D4 domain has five LRRs with one cysteine-rich domain on each end [47]. These domains together form a seven stranded β-sheet [47]. The interaction between D4 and HS is mediated by three highly positive basic patches at the bottom of the D4 [47]. Specifically, it has been shown that sulphate forms hydrogen bonds with the Tyr 810 and His 833 residues [47]. This is a highly conserved pattern across Slit domains [47]. The D4 domain is also involved in the dimerization of Slit [47]. It is believed that the Slit dimer, Robo, and cell-surface HS form a tetrameric complex [47].

HS’s pivotal involvement in the Slit/Robo interaction, and furthermore, Slit/Robo involvement in axonal guidance and dendritic spine formation, make this signaling pathway of interest when exploring the pathogenesis of autism. Robo1 and Robo2 has also been shown to be deficient in some cases of autism, suggesting that this pathway may be a possible mechanism by which HS deficiency could cause autism [37, 48]. Furthermore, in one study the Robo2 deficiency was associated with the anterior cingulate cortex, an area of the brain associated with social cognition [37]. Slit expression was found to be increased in ASD group in comparison to the control group [40]. In addition, a role for Slit/Robo malfunctioning in autism, dyslexia and other speech disorders is supported by a recent study showing that vocal learning birds (songbird, parrot and hummingbird) make a relatively rare connection between vocal learning circuits of the forebrain and brainstem vocal motor neurons, not seen in vocal nonlearning bird species or mice, mediated by specialized regulation of SLIT-ROBO genes [49]. The role of Slit/Robo in autism is clearly an important area for further study.

If Slit/Robo signaling leads to autism, does it do so through its role in axonal guidance or through its role in dendritic spine formation? Both changes in long-distance connections and dendritic spine morphology are present, so presumably either is possible. It seems that a connection to dendritic spines is more plausible, when you consider that several autism related disorders have been linked to dendritic spine dysgenesis [36]. Syndycan-2 also is expressed in high levels in dendritic spines and has been linked to spine maturation [22]. A female with mental retardation, multiple exostoses, and autism was shown to have an X;8 translocation, involved in the GRPR gene, and the SDC2 gene which codes Syndecan-2 [50]. This suggest that Syndecan-2 and dendritic spine dysgenesis are related to autism. Furthermore, it has been shown that Slit1 treated neurons had morphological changes in dendritic spines [39]. This suggest Slit plays a key role in dendritic spine formation.

## srGAPs

Slit/Robo regulates the formation of dendritic spines through an interaction with srGAPs (slit-robo GTPase Activating Proteins) [38, 51]. srGAPs are a family of small guanine triphosphatase activating proteins, which act on the Rho GTPase family [52]. Rho GTPase family members include Rac, Cdc42 and Rho, all of which are members of the RAS superfamily [53–55]. srGAPs are regulators of the actin cytoskeleton, which has been shown to be involved in the formation of growth cones of the neural cytoskeleton [38, 53, 55, 56]. There are three forms of srGAPs identified in humans: srGAP1, srGAP2 and srGAP3 [52]. srGAP1 lessens the formation of cellular protrusions, while srGAP2 and srGAP3 increase the formation of cellular protrusions [38, 57]. All the srGAPs are highly expressed in the brain, especially in the forebrain [52, 58]. All three srGAPs are mainly located in the neurons, but not the glia [58]. Robo1, srGAP1 and srGAP2 were found in the anterior SVZ, suggesting that the Robo1/srGAP interaction plays a role in either neurogenesis or the migration of newly formed neurons [52]. Of greater interest is the localization of srGAP1 in the nuclei of neurons in the cerebral cortex layers II–VI [58]. Abnormal increases in the densities of dendritic spines in ASD patients have been noted for these layers. In light of the role of srGAP1 in lessening cellular protrusions, autism may be linked to disturbances in this pathway. To answer this question, the processes of how srGAPs mediates dendritic spine ormation must be explored.

srGAPs has been found to be collocated with synapses and the heads of dendritic spines [59]. Immature spines are observed when srGAPs is inhibited [59]. Also KO mice had immature and more numerous spines [59]. srGAPs has been shown to interact with Robo during the process of dendritic spine formation [38, 59]. srGAP and Robo receptors have been found to be collocated in rat brain samples, suggesting that the srGAP/Robo relationship may mediate the dendritic spine formation [58]. Robo has also been shown to have an effect on dendritic spine formation [39].

Slit regulates this interaction between srGAPs and Robo [51]. To understand the srGAPs/Robo interaction we must first understand the structure of srGAPs. srGAPs consist of a RhoGAP domain, a SH3 domain, and a Fes/CIP4 homology (FCH) domain [52]. The Slit/Robo interaction is mediated by the SH3 domain of srGAPs and the proline-rich CC3 motif of Robo1 [52]. The SH3 site of srGAPs is contained in several different proteins and is involved in many signaling pathways [60]. The SH3 region has a hydrophobic cleft that acts as a recognition site. This hydrophobic cleft recognizes proteins that have a PXXP motif, in which X is usually arginine or a hydrophobic residue. This PXXP motif forms what is known as a collagen chain conformation. The hydrophobic cleft of SH3 interacts with Robo. Robo has four motifs: CC0, CC1, CC2, and CC3. CC3 is a proline rich motif that mediates Robo’s interaction with the PXXP recognition site of srGAPs. CC3 has a highly conserved sequence of TYTDDLPPPPVPPPAIKSP [PIR: Q1RMC8], which interacts with the hydrophobic cleft of SH3.

It is believed that Slit binds to Robo1 enhancing the ability of srGAP1 to bind to Robo1 [51]. This activates srGAP1 which inactivates Cdc42 (a Rho GTPase), which phosphates N-WASP and activates it [51]. Inactivating Cdc42, therefore decreases the activation of N-WASP, which decreases activation of Arp2/3 [51]. Arp2/3 promotes actin polymerization and its deactivation causes dendritic spine collapse [51]. In this way Slits repulsive effects may cause the collapse of dendritic spines. The malfunctioning of this pathway may increase dendritic spine formation, mediated by the increased activity of Arp2/3.

## Alternative mechanism

As noted earlier a disruption of Slit/Robo signaling could also lead to autism, by affecting axonal guidance. Disturbances of axonal guidance have been shown in valproic acid treated mice and postmortem ASD studies, suggesting that disruptions in axonal guidance may contribute to the etiology of autism [7, 9]. One problem with this mechanism is that Slit is a repulsive guidance cue, which leads to growth cone collapse. Axons in ASD make fewer long distance connections [7, 9]. The opposite would be expected if Slit/Robo signaling was reduced.

## Conclusion

Increased dendritic spine formation is a feature of autism [8]. The relationship between Slit/Robo and srGAPs with excess spine formation suggests a potential mechanism by which a HS deficiency can cause autism. The deficiency of HS could lead to a decrease in the interaction between Robo1 and Slit, and this in turn will decrease the binding of Robo1 and srGAP1. Ultimately, this will lead to an upstream increase in actin polymerization. This also suggests a mechanism by which decreased Robo1 could lead to autism. The Robo1 deficiency leads to decreased interaction with srGAP1, leading to upstream actin polymerization. Disruptions in this pathway could also be related to the presence of excess sAPPα in autistic brains [61]. sAPPα is linked to increased neurite formation and is also known to bind HS [62, 63]. It is possible that this binding could interfere with Slit/Robo signaling.

While the interaction of srGAPs with Slit/Robo has been investigated, it has not been investigated in a Robo deficient environment or HS deficient environment. An investigation of how these deficiencies affects the interaction of srGAPs with Slit/Robo and the downstream dendritic spine formation could provide insights into autism. It could be insightful to study the Slit/Robo activity of EXT KO mice to determine if this pathway is indeed affected by HS deficiency. Furthermore an investigation on Robo KO mice might help determine if this pathway effects dendritic spine morphology.

## Abbreviations

ASD: autism spectrum disorders
HS: heparan sulfate
srGAPs: slit/robo GTPase activating proteins
SVZ: sub-ventricular zone
LV-SVZ: lateral ventricle sub-ventricular zone
LRR: leucine-rich repeats
IG: immunoglobulin-like
CAM: cellular adhesion molecule
FCH: Fes/CIP4 homology
FISH: fluorescence in situ hybridization
NSCs: neural stem cells
USV: ultrasonic vocalizations
ECM: extra-cellular matrix
GlcA: glucuronic acid
H2ST: HS 2-O-sulfotransferases
HS C5-EP: heparan sulfate C5 epimerase
EXT: exostosin
GlcNAc: N-acetylglucosamine
UDPGDH: UDP glucose dehydrogenase
H6ST: 6-O-sulfotransferases
KO: knockout
